# Exploring Retene's Tumour‐Initiating Potential: Integrating Computational and Experimental Approaches

**DOI:** 10.1111/bcpt.70034

**Published:** 2025-04-10

**Authors:** Francisco Carlos da Silva Junior, Thiago Pires Cláudio, Ricardo Luiz Cavalcanti de Albuquerque‐Júnior, Silvia Regina Batistuzzo de Medeiros

**Affiliations:** ^1^ Department of Cell Biology and Genetics, Biosciences Center Federal University of Rio Grande do Norte Natal RN Brazil; ^2^ Graduate Program in Biochemistry and Molecular Biology, Biosciences Center Federal University of Rio Grande do Norte Natal RN Brazil; ^3^ Toxicology Centre University of Saskatchewan Saskatoon Canada; ^4^ Postgraduate Program in Dentistry, Health Sciences Center Federal University of Santa Catarina Florianópolis Santa Catarina Brazil; ^5^ Department of Pathology, Health Sciences Center Federal University of Santa Catarina Florianópolis Santa Catarina Brazil

**Keywords:** bioinformatic tools, carcinogenicity, chemical skin‐carcinogenesis, retene, Swiss albino mice

## 1 Introduction

Currently, the US Environmental Protection Agency (EPA) classifies 16 PAHs as priority pollutants, including seven carcinogenic compounds [1]. Nevertheless, numerous nonlisted PAHs may also contribute to carcinogenic effects, including Benzo[*a*]pyrene (B[*a*]P) and dibenzo[*a,h*]anthracene [2]. Retene (RET, 1‐methyl‐7‐isopropylphenanthrene) is a polycyclic aromatic hydrocarbon (PAH) primarily formed during the combustion of coniferous wood and is a significant component of atmospheric particulate matter from forest fires [3, 4]. Metabolic and mechanistic studies suggest that RET induces genotoxicity and chromosomal alterations through oxidative stress [3], raising concerns about its potential carcinogenicity. Although RET is structurally similar to well‐known carcinogenic PAHs, its toxicological risks remain poorly investigated.

Structure–activity relationship (SAR) models and in vivo studies are widely used to predict toxicity and assess carcinogenic potential [5]. PAHs are known to induce skin carcinogenesis via metabolic activation in dermal models [6], highlighting the relevance of such approaches. Given the limited data on RET's carcinogenic effects, this study aims to evaluate its tumour‐initiating potential using SAR predictions and an in vivo Swiss albino mouse model. To our knowledge, no previous studies have explored RET's involvement in carcinogenesis, and our findings could help clarify its underestimated toxicological impact.

## 2 Materials and Methods

### 2.1 In Silico Analysis

The in silico evaluation of RET's carcinogenic potential was performed using molecular structure‐based computational tools. Canonical SMILES sequences for RET (PubChem CID: 10222), benzo[*a*]pyrene (B[*a*]P; CID: 2336) and 7,12‐dimethylbenz[*a*]anthracene (DMBA; CID: 6001) were obtained from PubChem. B[*a*]P and DMBA were used as positive controls. Chemical structures of RET, B[*a*]P and DMBA can be found accessing the CID numbers at https://pubchem.ncbi.nlm.nih.gov/. Malignant cell transformation potential was assessed using Danish(Q)SAR (https://qsar.food.dtu.dk/), calibrated for Syrian hamster embryo (SHE) cells. Carcinogenicity in rodent models was predicted using PreADMET (https://preadmet.webservice.bmdrc.org/) and ToxTree v3.1 (https://toxtree.sourceforge.net/predict/), based on structural carcinogenicity alerts. Additionally, organ‐specific carcinogenicity was evaluated using ROSC‐pred (https://www.way2drug.com/rosc/), a PASS‐based tool comparing the molecular structure of RET with biologically active reference compounds, predicting activity probabilities (Pa) at thresholds of 30%, 50% and 70%.

### 2.2 In Vivo Analysis

#### 2.2.1 Animals, Chemicals and Dose Formulations

Forty‐two adult male Swiss albino mice (6–8 weeks old, 30 ± 5 g) were obtained from the Tiradentes University experimental vivarium and housed under controlled conditions (22 ± 1°C, 12‐h light/dark cycle). Water and a standard diet were provided ad libitum. Experimental protocols were approved by the institutional ethics committee (approval no. 010622) and adhered to ARRIVE guidelines [7] (Supporting Information S1). Moreover, the study was conducted in accordance with the Basic & Clinical Pharmacology & Toxicology policy for experimental and clinical studies [8].

Mice were randomly assigned to seven groups (*n* = 6) and treated with RET (10, 20, 30 and 40 μM; ChemService Inc.) via topical application (100 μL) on the shaved dorsal skin, following protocol by [9]. B[*a*]P and DMBA (10 μM each) served as positive controls, and an acetone‐only group was included as a solvent control. Acetone was selected for its rapid evaporation, facilitating direct tissue exposure to RET. Treatment followed an initiation–promotion regimen, applied three times weekly for 16 weeks. Mice were monitored for survival, body weight, and food/water intake throughout the study.

#### 2.2.2 Gross and Histomorphological Analysis

Skin lesions were classified into irritative (wrinkling, petechiae, hyperkeratosis, ulcerations ≤ 2 mm) or dysplastic/neoplastic changes (leukoplastic plaques, ulcerations >2 mm, papulonodular lesions). The injury index (InL) was calculated as follows: InL = (nL × nA)nT × 100InL (where *nL* is the number of lesions per animal, *nA* is the number of affected animals, and *nT* is the total animals per group). After 16 weeks, mice were euthanized via intraperitoneal ketamine:xylazine (30:300 mg/kg), and treated skin samples were excised, fixed in buffered formalin (10%), processed for paraffin embedding and sectioned (5 μm). Haematoxylin–eosin–stained slides were analysed microscopically. Histopathological classification followed established guidelines for cutaneous squamous cell carcinoma [10].

### 2.3 Statistical Analysis

Data normality and homoscedasticity were verified using Shapiro–Wilk and Levene tests. When normality and homoscedasticity were passed, parametric data (mean ± SD) were compared using ANOVA with Bonferroni post‐hoc analysis. Survival curves were analysed using the log‐rank (Mantel–Cox) test. Statistical significance was set at *p* < 0.05.

## 3 Results

According to Danish(Q)SAR calibrated for Syrian hamster embryo (SHE) cells (Table 1), RET, B[*a*]P and DMBA showed positive results for malignant transformation. Furthermore, structural alerts of the type *genotoxic carcinogenicity* were detected for all PAHs investigated using ToxTree (Table 1). Moreover, when organ‐specific carcinogenicity was analysed using the ROSC‐Pred calibrated for rodents, RET, B[*a*]P and DMBA were predicted to induce tumours in different organs or tissues, such as skin, lung, liver, and kidney (Supporting Information S2).

**TABLE 1:** Qualitative evaluation of transformation cell malignant and carcinogenic potential through structural alerts of carcinogenicity by the Danish(Q)SAR and Toxtree V 3.1 programs.

PAH	Danish(Q)SAR^a^	ToxTree V.3.1
	Structural alerts	
RET	Positive	Structural alert to genotoxic carcinogenicity
DMBA	Positive	Structural alert to genotoxic carcinogenicity
B[*a*]P	Positive	Structural alert to genotoxic carcinogenicity

^a^ The cell malignant transformation result is predicted in Syrian Hamster Embryo cells (SHE cells).

**FIGURE 1:**
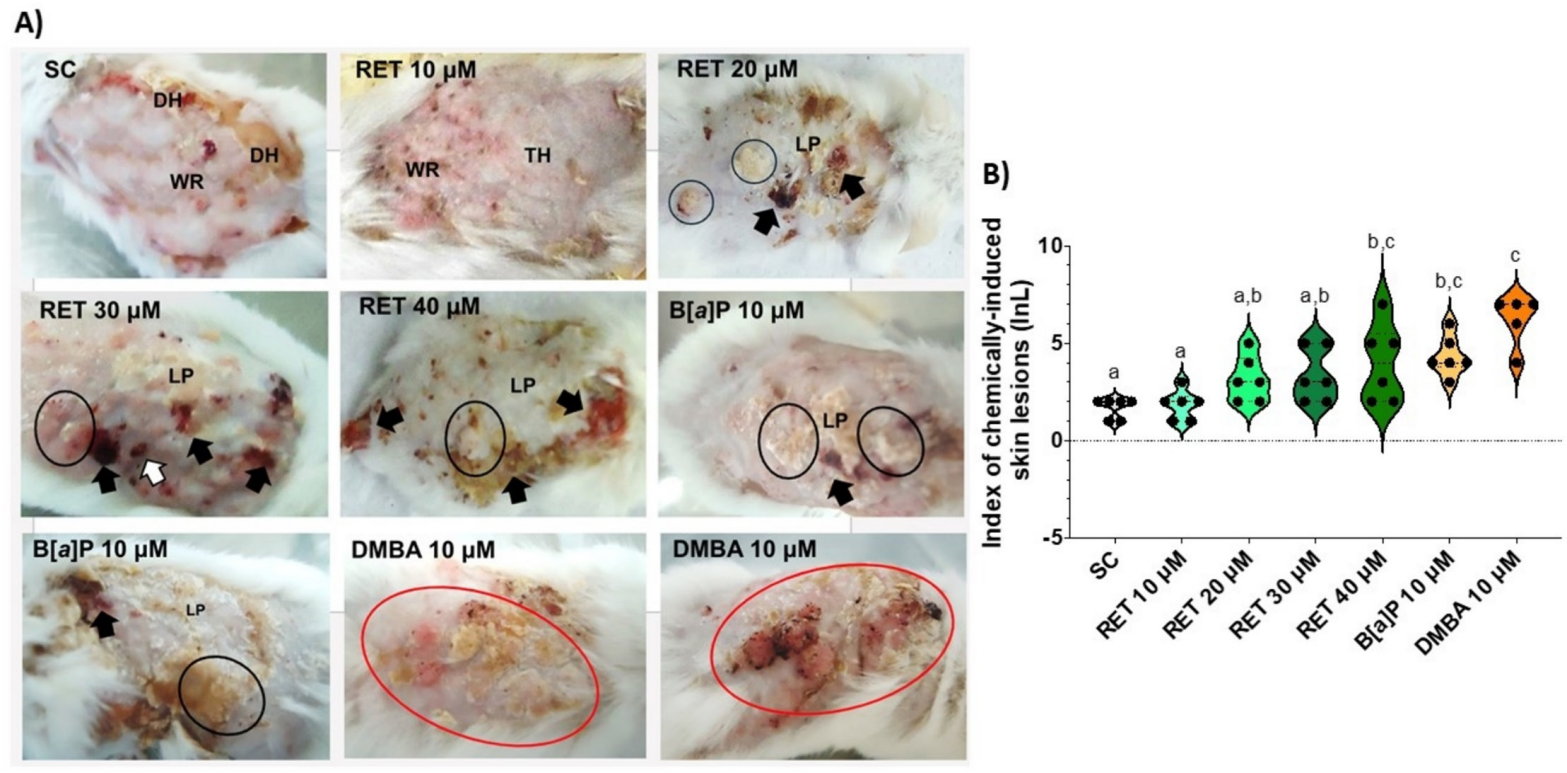
(A) Gross changes observed in the of the experimental animals. Skin wrinkling (WR) and thickening (TH), desquamative hyperkeratosis (DH), large (black arrow) and small (white arrow) skin ulcerations, leukoplastic plaques (LP), small isolated verrucous polypoid papules (area delimited by black line), as well as multiple and sometimes confluent, irregular polypoid nodules (area delimited by red line). (B) Assessment of the average index of chemically induced skin lesions (InL) in mice. Data are expressed as mean ± standard deviation (SD). Different letters above each column mean significantly different means (*p* < 0.05), whereas equal letters represent statistically similar means (*p* > 0.05) (ANOVA, followed by Bonferroni's multiple comparison test).

The compounds RET, B[*a*]P and DMBA did not induce changes in body weight (*F*_6,34_ = 1.203, *p* = 0.32) or increase mortality (*p* > 0.42) (Supporting Information S3). Water (*F*_15,90_ = 1.428, *p* = 0.15) and food intake (*F*_2,40_ = 0.904, *p* = 0.43) remained unchanged among the experimental groups (Supporting Information S3). After 16 weeks of chemical exposure (Figure 1A), the B[*a*]P, RET 10 μM and 30 μM groups developed papules, fungiform nodules and isolated leukoplastic plaques. In contrast, the SC and RET 10 μM groups exhibited skin thickening, wrinkling, minor ulcers and scaly hyperkeratosis, likely resulting from continuous acetone application. The InL varied significantly among groups (*F*_6,34_ = 8.601, *p* < 0.0001) (Figure 1B). The RET 40 μM group presented a significantly higher InL than SC (*p* = 0.03). In contrast, RET 20 and 30 μM displayed InL values comparable to B[*a*]P but significantly lower than DMBA (*p* = 0.005 and *p* = 0.009, respectively).

**FIGURE 2:**
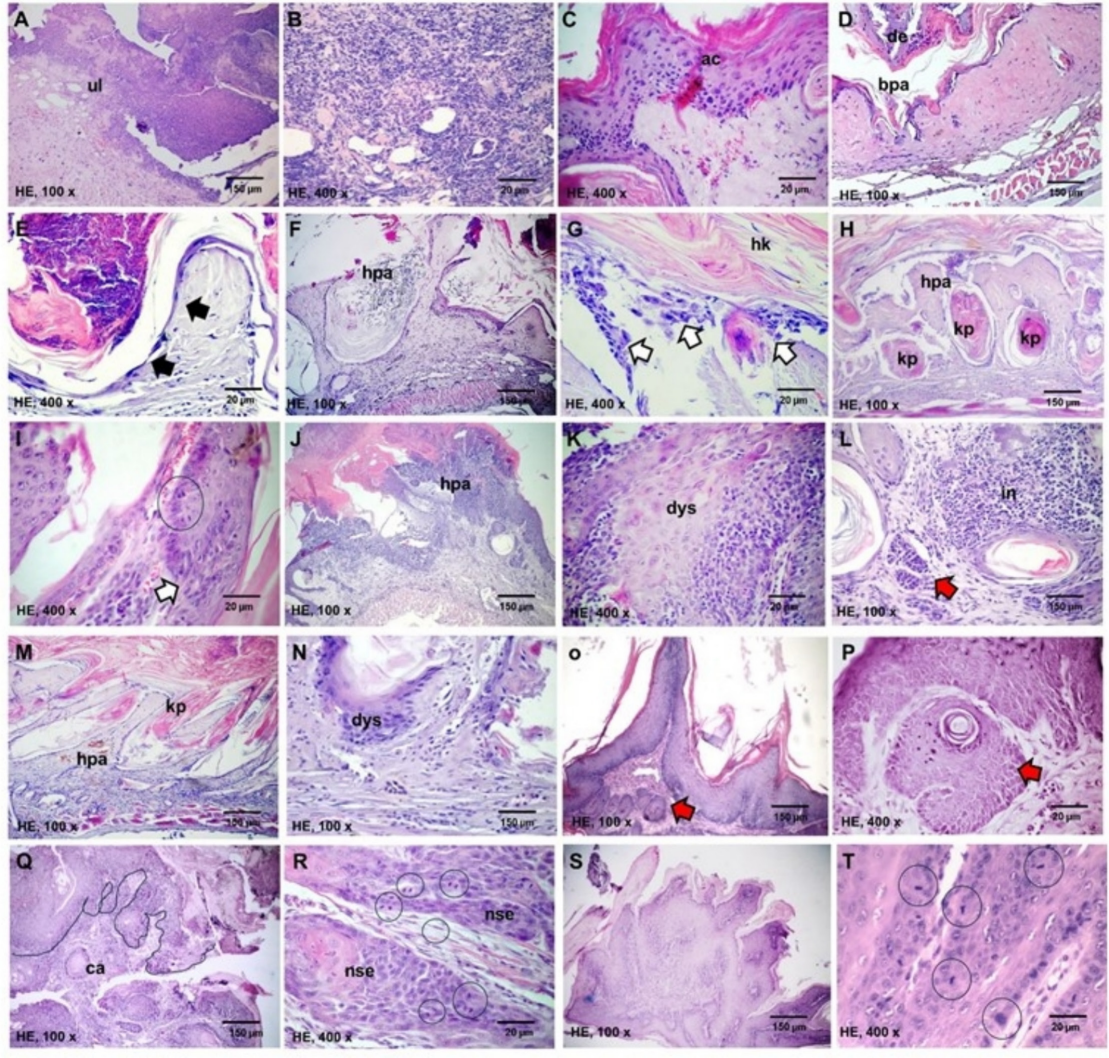
Photomicrographs of HE‐stained histological slides representative of the main histological changes observed in the skin of the experimental groups. (A–C) Areas of ulceration and marginal mild epithelial acanthosis in the SC group. (D,E) Group RET 10 μM shows focal areas of blunt papillomatosis with superficial epithelial desquamation and foci of epithelial atrophy. (F,G) Group RET 20 and (H,I) RET 30 μM showing higher dermal papillary projections covered by squamous epithelium with focal areas of mild dysplasia. In group RET 40 μM (J–L), high papillary projections covered by proliferative dysplastic epithelium. Scanty epithelial nests apparently ‘detached’ from the lining epithelium (pseudo carcinomatous hyperplasia) are observed in an intensely inflamed dermal connective tissue. Areas of papillary projections (M,N), covered by dysplastic hyperkeratinized squamous epithelium with keratin plugs formation; and (O,P) areas of development of markedly exophytic lesions with foci of pseudocarcinomatous hyperplasia. (Q,R) Invasive carcinoma deriving from the epidermal lining comprised of well‐differentiated neoplastic squamous cells; and (S,T) Noninvasive papillomas, with high mitotic index, observed in the DMBA‐treated animals.

Histologically (Figure 2), the SC group exhibited hyperkeratosis, epithelial atrophy, desquamative hyperkeratosis, ulceration and focal inflammation. Similar changes were observed in RET 10 μM, with occasional blunt exophytic papillae. The B[*a*]P 20 μM, RET 30 μM and RET 40 μM groups displayed prominent warty hyperkeratinized epitelial projections. Mild epithelial dysplasia and papillomas with foci os pseudoepitheliomatous hyperplasia were noted in B[*a*]P (66.6%) and RET 40 μM (83.3%). Invasive squamous cell carcinoma developed only in DMBA‐treated specimens (60%), whereas papilloma with increased mitotic activity and epithelial dysplasia were identified in the remaining 40% of cases.

## 4 Discussion

The mouse skin initiation‐promotion model is a well‐established tool for assessing the carcinogenic potential of polycyclic aromatic hydrocarbons (PAHs). It provides standardized and reproducible data with clear endpoints [11]. Unlike systemic models, dermal exposure allows localized treatment, reducing systemic toxicity and aligning with ethical considerations. Regulatory agencies utilize these data to evaluate PAH potency and associated carcinogenic risks in humans [12]. This study is the first to suggest that RET may play a role in carcinogenesis, as supported by both in silico and in vivo findings.

The high lesion index (InL) observed in the RET 40 μM, B[*a*]P 10 μM and DMBA 10 μM groups suggest that RET may contribute to skin carcinogenesis. Histopathological evaluation revealed epithelial dysplasia and focal infiltrative epithelial proliferation in RET 40 μM–treated animals, consistent with chemical carcinogenesis. Additionally, RET‐induced oxidative stress reinforces its genotoxic potential [3], as reactive metabolites can interact with DNA, leading to mutations. Previous studies have demonstrated RET's genotoxicity in vitro and zebrafish models [3, 13], while in silico analysis further supports its involvement in skin cancer development. Oxidative stress, a hallmark of PAH exposure, is known to compromise antioxidant defences and promote chronic inflammation via the upregulation of pro‐inflammatory cytokines such as TNF‐α, IL‐1β and IL‐6, thereby contributing to tumorigenesis [14]. The pronounced inflammatory response observed in RET 40 μM‐treated animals reinforces the critical role of inflammation in carcinogenic progression.

PAHs, including B[*a*]P and DMBA, promote carcinogenesis through metabolic activation and epigenetic modifications [6]. RET may follow a similar mechanism, particularly during the promotion phase of PAH‐induced skin carcinogenesis. This phase involves key gene mutations in epidermal keratinocytes, primarily mediated by cytochrome P450 enzyme metabolites [10]. While B[*a*]P and DMBA undergo metabolic conversion to highly reactive diol epoxide intermediates; RET is metabolized into ortho‐quinones, which can form DNA adducts and disrupt replication fidelity.

Histological analysis revealed that B[*a*]P 10 μM and RET 40 μM exposure predominantly induced papillomatous epithelial tumours, whereas DMBA 10 μM exposure resulted in invasive squamous cell carcinomas. This suggests that RET may preferentially promote nonmalignant or exophytic tumours rather than aggressive invasive variants. Differences in tumour histopathology between RET and DMBA exposure imply distinct underlying mechanisms of carcinogenesis, potentially involving variations in oncogenic pathway activation, cellular receptor interactions or the ability to induce inflammation and tissue remodelling. These findings highlight the need for further investigation into the molecular mechanisms underlying RET‐induced proliferative effects and its potential role as a tumour promoter, particularly in environmental exposure to PAHs.

Study limitations include reliance on structure‐based predictive models, the use of single‐sex and strain and a study duration of fewer than 18 months, which may limit extrapolation to human risk. Additionally, molecular and biochemical analyses were not performed, as the study primarily focused on histopathological endpoints. However, including well‐characterized carcinogenic PAHs provides a robust comparative framework for evaluating RET's biological effects.

In summary, this study suggests that RET, an environmental contaminant, may possess both tumour‐initiating and tumour‐promoting properties, as demonstrated by computational modelling and in vivo experimentation. Further research is needed to elucidate its toxicokinetics and potential implications for human carcinogenic risk, particularly concerning respiratory exposure from forest fire emissions.

### Conflicts of Interest

The authors declare no conflicts of interest.

## Supporting information


**Data S1** Supporting Information

**Table S2** Specific carcinogenicity predicted by the ROSC‐pred of RET, B[*a*]P, and DMBA.

**Figure S1** A) Body weight (in grams) in *Mus musculus* mice treated with RET 10 μM, 20 μM, 30 μM and 40 μM diluted in acetone. Positive controls (PC) are the chemicals DMBA at 10 μM and B[*a*]P at 10 μM were used. As a solvent control (SC), only P.A. acetone solution was used as vehicle. Mice were treated for 16 weeks. No significant differences were found between the groups according to ANOVA test following the Bonferroni multiple comparison *post‐hoc* test. B) Survival curves for mice treated with doses of RET, B[*a*]P, and DMBA.
**Figure S2**: Intake of water (mL/day) in *Mus musculus* mice treated with RET 10, 20, 30, and 40 μM diluted in acetone. Positive controls (PC) are the chemicals DMBA at 10 μM and B[*a*]P at 10 μM were used. As a solvent control (SC), only P.A. acetone solution was used as vehicle. Mice were treated for 16 weeks. No significant differences were found between the groups according to ANOVA test following the Bonferroni multiple comparison *post‐hoc* test.
**Figure S3**: Intake of food (mg/day) in *Mus musculus* mice treated with RET 10, 20, 30, and 40 μM diluted in acetone. Positive controls (PC) are the chemicals DMBA at 10 μM and B[*a*]P at 10 μM were used. As a solvent control (SC), only P.A. acetone solution was used as vehicle. Mice were treated for 16 weeks. No significant differences were found between the groups according to ANOVA test following the Bonferroni multiple comparison *post‐hoc* test.

## Data Availability

The data that supports the findings of this study are available in the Supporting Information of this article.
