# Supplementary material for: Exploring Retene's Tumour‐Initiating Potential: Integrating Computational and Experimental Approaches
Source: Basic Clin Pharmacol Toxicol. 2025 Apr 10;136(5):e70034. doi: 10.1111/bcpt.70034 (PMC11985699; doi:10.1111/bcpt.70034)
Supplement: Supplementary file 2 — Table S2 Specific carcinogenicity predicted by the ROSC‐pred of RET, B[a]P, and DMBA. [file BCPT-136-0-s003.docx]

**Supplementary material S2**

**Supplementary Table S2**: Specific carcinogenicity predicted by the ROSC-pred of RET, B[*a*]P, and DMBA.

| **PAH** | **Mouse** | | | | | | **Rats** | | | | | |
| --- | --- | --- | --- | --- | --- | --- | --- | --- | --- | --- | --- | --- |
|  | **Male** | | | **Female** | | | **Male** | | | **Female** | | |
|  | Pa^1^ | Pi^2^ | Organ | Pa^1^ | Pi^2^ | Organ | Pa^1^ | Pi^2^ | Organ | Pa^1^ | Pi^2^ | Organ |
| RET | 0.728  0.589  0.418  0.386  0.121 | 0.064  0.081  0.235  0.233  0.015 | Urinary bladder  Lung  Thyroid gland  Vascular system  Small intestine | 0.780  0.751  0.673  0.597  0.462  0.447  0.225  0.385  0.121 | 0.031  0.050  0.079  0.159  0.111  0.105  0.071  0.258  0.015 | Stomach  Ovary  Urinary bladder  Thyroid gland  Vascular system  Skin  Peritoneal cavity  Liver  Small intestine | 0.658  0.693  0.520  0.454  0.271  0.418  0.484  0.407  0.265  0.340 | 0.049  0.207  0.174  0.143  0.027  0.177  0.249  0.178  0.178  0.306 | Ear zymbals gland  Kidney  Thyroid gland  Spleen  Skin  Nasal cavity  Hematopoietic system  Oral cavity  Lung  Urinary bladder | 0.644  0.562  0.620  0.541  0.338  0.346  0.413  0.331  0.355  0.406  0.332 | 0.056  0.054  0.148  0.200  0.054  0.145  0.248  0.225  0.258  0.258  0.287 | Ear zymbals gland  Mammary gland  Hematopoietic system  Uterus  Skin  Nasal cavity  Urinary bladder  Lung  Thyroid gland  Kidney  Liver |

**^1^** Probability of being active; **^2^** Probability of being inactive.

**Supplementary Table S2**: Specific carcinogenicity predicted by the ROSC-pred of RET, B[*a*]P, and DMBA.

| **PAH** | **Mouse** | | | | | | **Rats** | | | | | |
| --- | --- | --- | --- | --- | --- | --- | --- | --- | --- | --- | --- | --- |
|  | **Male** | | | **Female** | | | **Male** | | | **Female** | | |
|  | Pa^1^ | Pi^2^ | Organ | Pa^1^ | Pi^2^ | Organ | Pa^1^ | Pi^2^ | Organ | Pa^1^ | Pi^2^ | Organ |
| B[*a*]P | 0.728  0.589  0.418  0.386  0.121 | 0.064  0.081  0.235  0.233  0.015 | Urinary bladder  Lung  Thyroid gland  Vascular system  Small intestine | 0.780  0.751  0.673  0.597  0.462  0.447  0.225  0.385  0.121 | 0.031  0.050  0.079  0.159  0.111  0.105  0.071  0.258  0.015 | Stomach  Ovary  Urinary bladder  Thyroid gland  Vascular system  Pituitary gland  Peritoneal cavity  Liver  Small intestine | 0.658  0.693  0.520  0.454  0.271  0.418  0.484  0.407  0.265  0.340 | 0.049  0.207  0.174  0.143  0.027  0.177  0.249  0.178  0.178  0.306 | Ear zymbals gland  Kidney  Thyroid gland  Spleen  Maltoni head cancers  Nasal cavity  Hematopoietic system  Oral cavity  Lung  Urinary bladder | 0.644  0.562  0.620  0.541  0.338  0.346  0.413  0.331  0.355  0.406  0.332 | 0.056  0.054  0.148  0.200  0.054  0.145  0.248  0.225  0.258  0.329  0.287 | Skin  Mammary gland  Hematopoietic system  uterus  Clitoral gland  Nasal cavity  Urinary bladder  Lung  Thyroid gland  Kidney  Liver |

**^1^** Probability of being active; **^2^** Probability of being inactive.

**Supplementary Table S2:** Specific carcinogenicity predicted by the ROSC-pred of RET, B[*a*]P, and DMBA (*continued*).

| **PAH** | **Mouse** | | | | | | **Rats** | | | | | |
| --- | --- | --- | --- | --- | --- | --- | --- | --- | --- | --- | --- | --- |
|  | **Male** | | | **Female** | | | **Male** | | | **Female** | | |
|  | Pa^1^ | Pi^2^ | Organ | Pa^1^ | Pi^2^ | Organ | Pa^1^ | Pi^2^ | Organ | Pa^1^ | Pi^2^ | Organ |
| DMBA | 0.821  0.598  0.633  0.454  0.434  0.440  0.153 | 0.026  0.074  0.128  0.152  0.185  0.266  0.005 | Urinary Bladder  Lung  Thyroid gland  Vascular system  Liver  Stomach  Small intestine | 0.755  0.743  0.741  0.692  0.566  0.510  0.470  0.296  0.436  0.153 | 0.049  0.039  0.048  0.092  0.061  0.052  0.171  0.041  0.222  0.005 | Ovary  Urinary bladder  Stomach  Thyroid gland  Vascular system  Pituitary gland  Liver  Peritoneal cavity  Lung  Small intestine | 0.741  0.616  0.592  0.576  0.588  0.551  0.500  0.330  0.487  0.450  0.346  0.393  0.377  0.270 | 0.163  0.087  0.091  0.080  0.133  0.105  0.105  0.011  0.197  0.176  0.144  0.210  0.261  0.166 | Kidney  Thyroid gland  Spleen  Ear zymbals gland  Hematopoietic system  Oral cavity  Nasal cavity  Maltoni head cancers  Liver  Urinary bladder  Skin  All tumor-bearing animals  Stomach  Lung | 0.616  0.609  0.627  0.518  0.507  0.474  0.418  0.340  0.420  0.356  0.340  0.325  0.224 | 0.068  0.120  0.138  0.130  0.163  0.145  0.092  0.053  0.152  0.112  0.214  0.204  0.160 | Ear zymbals gland  Uterus  Hematopoietic system  Urinary bladder  Kidney  Liver  Nasal cavity  Clitoral gland  Thyroid gland  Mammary gland  Lung  All tumor-bearing animals  Oral cavity |

**^1^** Probability of being active; **^2^** Probability of being inactive.

**References**

[1] I.V. Ferrari, Open access in silico tools to predict the ADMET profiling and PASS (Prediction of Activity Spectra for Substances of Bioactive compounds of Garlic (Allium sativum L.), BioRxiv (2021) 2021.07.18.452815. https://doi.org/10.1101/2021.07.18.452815.

[2] A. Lagunin, A. Rudik, D. Druzhilovsky, D. Filimonov, V. Poroikov, ROSC-Pred: web-service for rodent organ-specific carcinogenicity prediction, Bioinformatics 34 (2018) 710–712. https://doi.org/10.1093/bioinformatics/btx678.
