# Supplementary material for: Exploring Retene's Tumour‐Initiating Potential: Integrating Computational and Experimental Approaches
Source: Basic Clin Pharmacol Toxicol. 2025 Apr 10;136(5):e70034. doi: 10.1111/bcpt.70034 (PMC11985699; doi:10.1111/bcpt.70034)
Supplement: Supplementary file 3 — Figure S1 A) Body weight (in grams) in Mus musculus mice treated with RET 10 μM, 20 μM, 30 μM and 40 μM diluted in acetone. Positive controls (PC) are the chemicals DMBA at 10 μM and B[a]P at 10 μM were used. As a solvent control (SC), only P.A. acetone solution was used as vehicle. Mice were treated for 16 weeks. No significant differences were found between the groups according to ANOVA test following the Bonferroni multiple comparison post‐hoc test. B) Survival curves for mice treated with doses of RET, B[a]P, and DMBA. Figure S2: Intake of water (mL/day) in Mus musculus mice treated with RET 10, 20, 30, and 40 μM diluted in acetone. Positive controls (PC) are the chemicals DMBA at 10 μM and B[a]P at 10 μM were used. As a solvent control (SC), only P.A. acetone solution was used as vehicle. Mice were treated for 16 weeks. No significant differences were found between the groups according to ANOVA test following the Bonferroni multiple comparison post‐hoc test. Figure S3: Intake of food (mg/day) in Mus musculus mice treated with RET 10, 20, 30, and 40 μM diluted in acetone. Positive controls (PC) are the chemicals DMBA at 10 μM and B[a]P at 10 μM were used. As a solvent control (SC), only P.A. acetone solution was used as vehicle. Mice were treated for 16 weeks. No significant differences were found between the groups according to ANOVA test following the Bonferroni multiple comparison post‐hoc test. [file BCPT-136-0-s001.docx]

**Supplementary material S3**

**
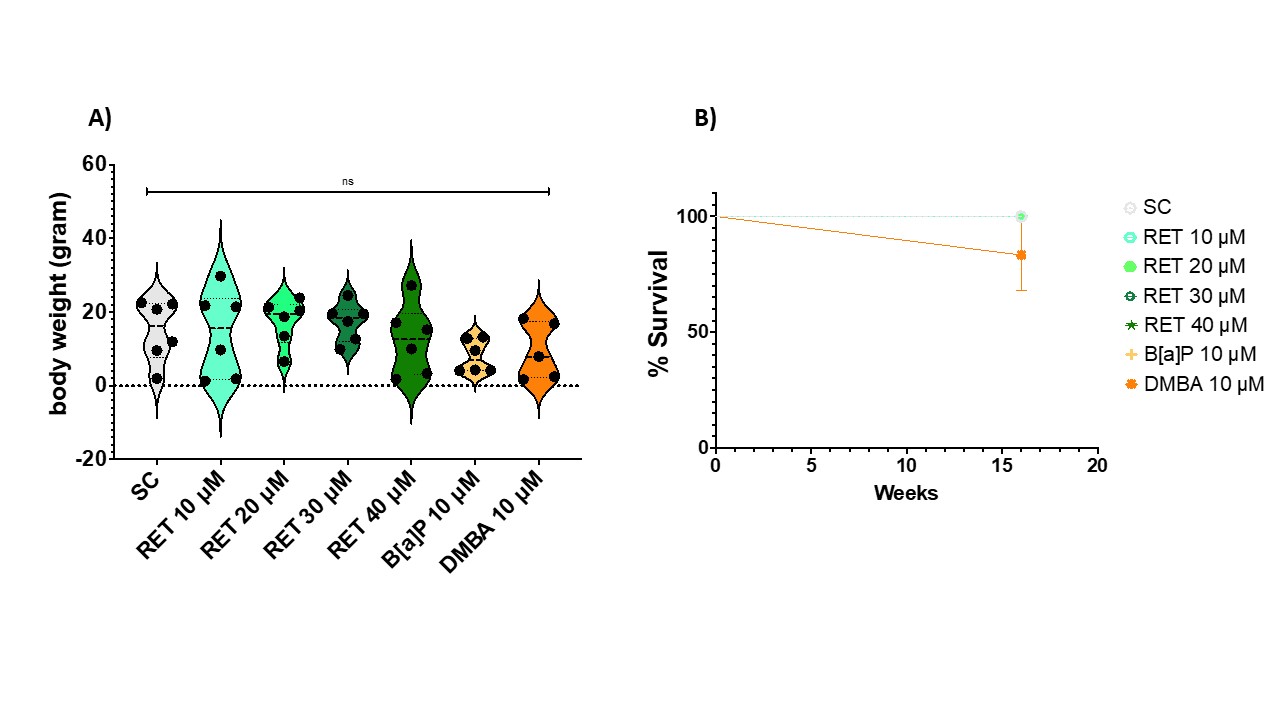
**

**Figure S1**: A) Body weight (in grams) in *Mus musculus* mice treated with RET 10 µM, 20 µM, 30 µM and 40 µM diluted in acetone. Positive controls (PC) are the chemicals DMBA at 10 µM and B[*a*]P at 10 µM were used. As a solvent control (SC), only P.A. acetone solution was used as vehicle. Mice were treated for 16 weeks. No significant differences were found between the groups according to ANOVA test following the Bonferroni multiple comparison *post-hoc* test. B) Survival curves for mice treated with doses of RET, B[*a*]P, and DMBA.


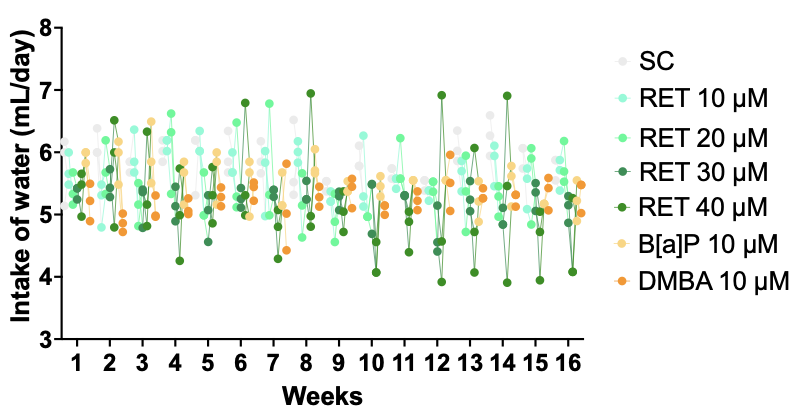
**Figure S2**: Intake of water (mL/day) in *Mus musculus* mice treated with RET 10, 20, 30, and 40 μM diluted in acetone. Positive controls (PC) are the chemicals DMBA at 10 μM and B[*a*]P at 10 μM were used. As a solvent control (SC), only P.A. acetone solution was used as vehicle. Mice were treated for 16 weeks. No significant differences were found between the groups according to ANOVA test following the Bonferroni multiple comparison *post-hoc* test.


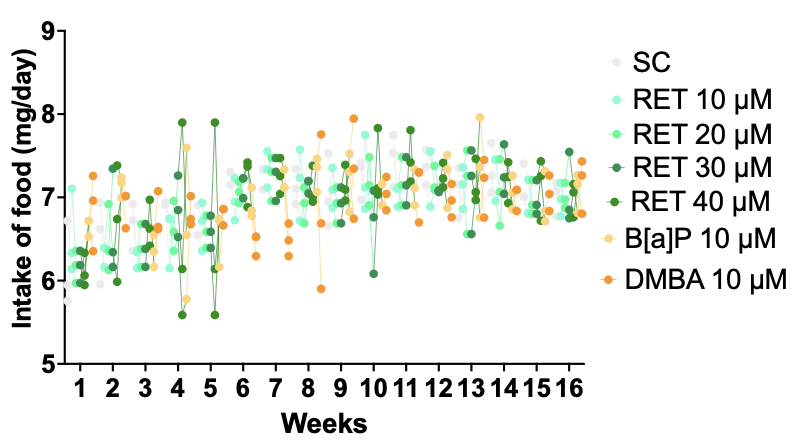


**Figure S3**: Intake of food (mg/day) in *Mus musculus* mice treated with RET 10, 20, 30, and 40 μM diluted in acetone. Positive controls (PC) are the chemicals DMBA at 10 μM and B[*a*]P at 10 μM were used. As a solvent control (SC), only P.A. acetone solution was used as vehicle. Mice were treated for 16 weeks. No significant differences were found between the groups according to ANOVA test following the Bonferroni multiple comparison *post-hoc* test.
